# The Relationship between Phase Angle, Nutrition Status, and Complications in Patients with Pancreatic Head Cancer

**DOI:** 10.3390/ijerph19116426

**Published:** 2022-05-25

**Authors:** Shengnan Zhou, Zhangping Yu, Xiaodong Shi, Huaiyu Zhao, Menghua Dai, Wei Chen

**Affiliations:** 1 Department of General Surgery, State Key Laboratory of Complex Severe and Rare Diseases, Peking Union Medical College Hospital, Chinese Academy of Medical Sciences & Peking Union Medical College, Beijing 100730, China; pumc_zhousn@student.pumc.edu.cn (S.Z.); pumc_yuzp@student.pumc.edu.cn (Z.Y.); 2 Department of Clinical Nutrition, State Key Laboratory of Complex Severe and Rare Diseases, Peking Union Medical College Hospital, Chinese Academy of Medical Sciences & Peking Union Medical College, Beijing 100730, China; sunnystation@126.com (X.S.); zhy65929@sina.com (H.Z.)

**Keywords:** phase angle, pancreatic head cancer, nutrition status, complications, bioelectrical impedance analysis

## Abstract

Phase angle (PhA), a bioimpedance parameter, is used to assess the nutrition status and body composition of patients. Patients with pancreatic head cancer often present with body composition changes that relate to adverse outcomes. PhA may be useful to evaluate prognosis in these patients, but data are deficient. We aim to explore the effects of PhA on nutrition evaluation and short-term outcome prediction in these patients. This prospective study included 49 participants with pancreatic head cancer who underwent pancreaticoduodenectomy (PD). All participants’ nutritional status and postoperative complications were assessed using nutrition assessment tools and the Clavien–Dindo classification method, respectively. Spearman correlation analyses were used to evaluate the association between PhA, nutrition status, and postoperative complications. ROC curves were generated to evaluate the ability of PhA to predict malnutrition and complications and to determine the cutoff value. The PhA values of the nutritional risk group and the malnourished group were significantly lower than those of the well-nourished group (*p* < 0.05). PhA positively correlated with patients’ nutrition status. Nineteen patients had postoperative complications, and the PhA value of the complication group was significantly lower than that of the non-complication group (4.94 vs. 5.47, *p* = 0.013). ROC curves showed that the cutoff point of PhA to predict malnutrition was 5.45 (AUC: 0.744), and the cutoff point of PhA to predict postoperative complications was 5.35 (AUC: 0.717). Our study indicates that PhA was associated with nutrition status and could be considered a nutrition assessment tool for pancreatic head cancer patients and predict the postoperative complications of these patients who have undergone PD.

## 1. Introduction

Pancreatic cancer is one of the most common malignant tumors of the digestive system, and it is the most fatal cancer [1]. Patients with pancreatic head tumors more easily suffer from malnutrition at the time of diagnosis [2], and approximately 52–88% of pancreatic patients who undergo surgery are identified as having a moderate to severe risk of malnutrition [3]. Malnutrition has been recognized as an important risk factor for surgical outcomes. Thus, it is important to identify patients’ nutrition status before surgery.

A nutritional risk screening is usually recommended for hospitalized cancer patients, using the Nutritional Risk Screening 2002 (NRS-2002) score [4] and the Subjective Global Assessment (SGA) score [5] to assess whether the patient is malnourished. Recently, the nutrition association approved the latest standards for diagnosing malnutrition as follows: the Global Leadership Initiative on Malnutrition (GLIM) tool [6]. The differences among nutrition status assessment tools [7] impact the identification and interpretation of the nutrition status of cancer patients from different populations, thus affecting early intervention in cancer patients.

Measurements of body composition as an objective nutritional assessment method have been commonly used in recent years [8], and bioelectrical impedance analysis (BIA) [9,10] is widely used to measure body composition and assess the nutritional status of patients due to its advantages of being simple, inexpensive, and noninvasive. The phase angle (PhA) [11] is a parameter that can indicate the relationship between resistance (R) and capacitive reactance (Xc) generated from BIA devices: PhA = arctan(Xc/R) × 180/π.

PhA is thought to reflect cellular integrity; the larger the PhA value, the more intact the cell membrane and the better the cell function [12]. Because of this characteristic, PhA has been suggested as a nutrition screening tool for patients with conditions such as liver cirrhosis [13], and some studies have even reported that low PhA is related to postoperative complications in cancer patients [14]. However, the cutoff point values of PhA for clinical assessment are different, which may be caused by differences in disease physiology, race, and measurement devices. To date, no published studies have reported the value of PhA in patients with pancreatic head cancer who received pancreaticoduodenectomy (PD).

Thus, we aim to assess the association between PhA values and the nutrition status of pancreatic head cancer patients that were evaluated by different tools, such as the NRS-2002, SGA, and GLIM. In addition, the possible associations between PhA, nutrition status, and complications were also evaluated to determine the nutrition-related indicators that have the potential ability to predict the incidence of complications.

## 2. Methods

### 2.1. Subjects

**Figure 1:**
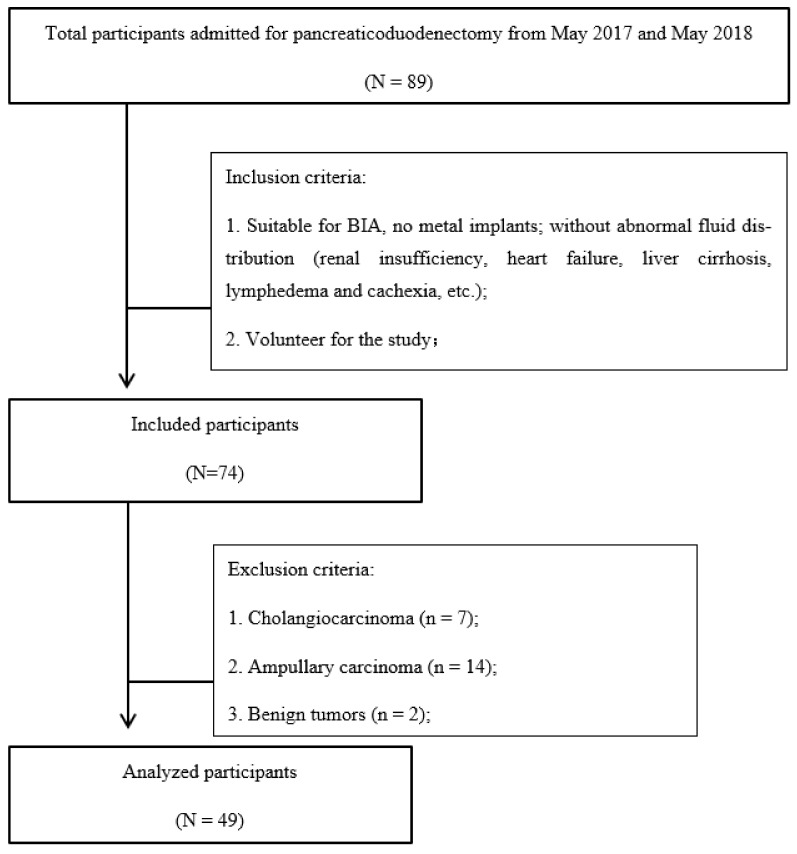
Selection of participants analyzed in this study. BIA, bioelectrical impedance analysis.

This prospective study included 74 participants diagnosed with space-occupying lesions of the head of the pancreas admitted for pancreaticoduodenectomy surgery at the General Surgery Department in the Peking Union Medical College Hospital (PUMCH) between May 2017 and May 2018. We applied the following patient inclusion criteria: those with pancreatic cancer confirmed by pathology; those who underwent laparoscopic or open pancreaticoduodenectomy surgery; and those able or willing to give informed consent. We excluded 7 participants diagnosed with cholangiocarcinoma through the postoperative paraffin pathology, 14 with ampullary carcinoma, 2 with benign tumors, and 2 with metachronous metastatic cancer. Finally, we collected and analyzed data from 49 participants (Figure 1). Clinical data, such as age, sex, BMI, and serum albumin (ALB), were recorded. This study was approved by the medical ethics committee of PUMCH, approval No. NCT02831725. The study was conducted in accordance with the ethical standards of the Helsinki Declaration of 1975.

### 2.2. Nutritional Assessment

Nutritional assessment was performed within 1 day of participants being hospitalized by a professional clinical nutritionist according to the NRS-2002, SGA, and GLIM methods, and the results were recorded on a case report form.

The NRS-2002 is a commonly used validated nutritional risk assessment score for hospitalized patients. It consists of a nutritional score, disease severity score, and age adjustment for patients aged >70 years (+1). The NRS score is the total of the nutritional score, disease severity score, and age adjustment. When the score is greater than or equal to 3 points, the risk of malnutrition is considered, and a score of less than three points indicates no malnutrition risk.

The SGA score includes the patient’s medical history, physical examination results, and the overall judgment of the clinician regarding the patient’s condition. Patients are classified as well-nourished when SGA is graded A and malnourished when SGA is graded B or C.

All hospitalized patients with a risk of malnutrition underwent the GLIM malnutrition diagnosis process. The process includes analyzing the case data and body composition data of patients at risk of malnutrition according to two criteria for a malnutrition diagnosis: type criteria and etiological criteria. Patients with a definitive diagnosis of malnutrition were classified as having first-stage (moderate) malnutrition or second-stage (severe) malnutrition based on the threshold of the severity of the patient’s malnutrition.

### 2.3. Anthropometric and Bioelectrical Impedance Analysis Measurements

The participant’s weight was measured on a mechanical platform scale to the nearest 0.1 kg, and height was measured using a stadiometer to the nearest 1 cm. BMI was derived as weight (kg) divided by height (m) squared (kg/m^2^). BIA for all participants was assessed using the eight-electrode mode with the Inbody S10 system (Biospace, Gyeonggi-do, Korea), with the patient in the supine position, arms and legs apart, in the morning before breakfast (after fasting overnight) and wearing uniform thin cotton clothes. The PhA, total body water (TBW), skeletal muscle, fat-free mass (FFM), fat mass (FM), and biceps circumference were recorded.

### 2.4. Clinical Outcomes

Postoperative complications were determined according to the definitions for postoperative complications from the “Bulletin of the American College of Surgeons” [15]. The diagnosis and grading of postoperative complications were performed with reference to the Clavien–Dindo criteria [16]. The length of hospital stay was determined as the number of days between admission and discharge. In addition, expenses during hospitalization were also recorded.

### 2.5. Statistical Analysis

The data analysis was performed using SPSS Version 25 (SPSS Inc., Chicago, IL, USA). In the descriptive statistics, the continuous variables are expressed as the mean ± standard deviation. The differences between groups were determined by the independent-samples *t*-test and chi-square test. A receiver operating characteristic (ROC) curve was used to determine the PhA cutoff values, and Spearman correlation analysis was used to analyze the relationship between PhA and the nutrition status and postoperative complications. A value of *p* < 0.05 was considered significant for all the tests.

## 3. Results

### 3.1. Patient Characteristics

A total of 49 pancreatic cancer patients who underwent PD were eligible for the study. The enrolled participants had a mean age of 58.08 ± 11.26 years, and 61% were men. Regarding the results of the BIA analysis, the value of PhA of female participants was 4.97 ± 0.70, which was significantly lower than the value of male participants (5.44 ± 0.73, *p* = 0.035). In addition, other body composition parameters, such as TBW, skeletal muscle, and FFM, of the female group were significantly lower than those of the male group (*p* < 0.05). Table 1 summarizes the participants’ characteristics prior to the operation. Among the patients with pancreatic head cancer, we observed significant negative correlations between PhA and age (*p* = 0.005), and positive correlations between PhA and BMI (*p* = 0.023), biceps circumference (*p* = 0.003) and ALB (*p* = 0.02), which are presented in Table 2.

### 3.2. Association between PhA and Nutritional Status

**Figure 2:**
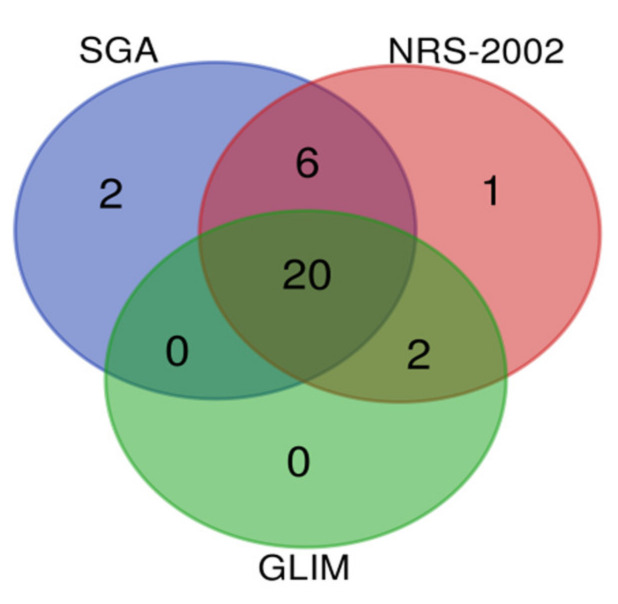
Venn diagram about the number of participants with malnutrition status according to the different nutritional status assessment methods.

The nutritional status of participants varied according to the nutrition assessment tool used. First, the results showed that 29 participants (59%) were at risk of malnutrition according to the NRS-2002 tool. In addition, 22 out of 29 participants were diagnosed with malnutrition with the GLIM criteria; among them, 8 participants had moderate malnutrition, and 14 participants had severe malnutrition. Next, according to the SGA score, 28 participants (57%) were at risk of malnutrition. These patients had some overlap, but were not identical (Figure 2). In addition, according to the sarcopenia cutoff points proposed by the European Working Group on Sarcopenia in Older People (EWGSOP) [17], only three participants met the diagnostic criteria for sarcopenia. The PhA values of participants with malnourished status were significantly lower than those of well-nourished participants, and the difference was statistically significant (*p* < 0.05, Table 3). Spearman correlation analysis showed that PhA was significantly correlated with the nutritional status of the patient, as shown in Table 4.

**Figure 3:**
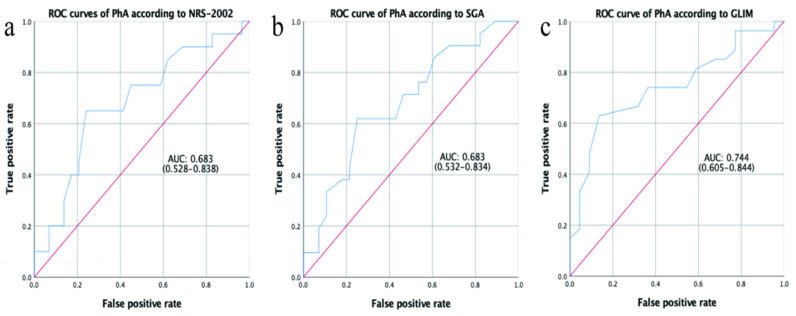
ROC curves of PhA to predict the patient nutrition status according to Nutritional Risk Screening 2002 (NRS-2002, (**a**)), Subjective Global Assessment (SGA, (**b**)), and Global Leadership Initiative on Malnutrition (GLIM, (**c**)). AUC: Area under the curve.

To determine the cutoff value of PhA for assessing patient nutrition status, we performed a ROC analysis according to the NRS-2002, SGA, and GLIM methods. The area under the ROC analysis of the PhA (AUC) was 0.744 (*p* = 0.004) according to GLIM, which was larger than that of NRS-2002 (0.683, *p* = 0.031) and SGA (0.683, *p* = 0.03), and suggested that PhA has the potential ability to predict malnutrition. The ROC curves for PhA in predicting malnutrition status in pancreatic head cancer patients are shown in Figure 3. According to the GLIM assessment, the cutoff value of PhA for diagnosing malnutrition was 5.45, with 63% sensitivity and 86.4% specificity (Youden index: 0.494). A PhA value below 5.45 was considered a low value, and a lower PhA indicated malnutrition status.

### 3.3. Association between PhA, Nutritional Status, and Complications

**Figure 4:**
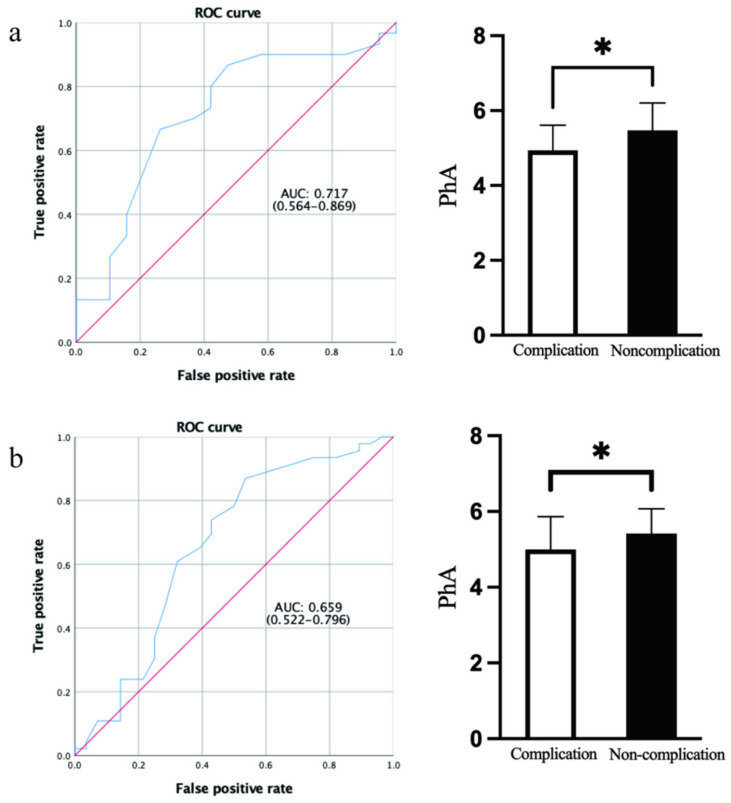
ROC curves of PhA to predict the postoperative complications and bar graphs of PhA values between the complication group and the non-complication group. (**a**) The results of 49 participants with pancreatic head cancer. (**b**) The results of 74 participants who underwent pancreaticoduodenectomy. * *p* < 0.05.

Among the 49 participants diagnosed with pancreatic head cancer in the present study, a total of 19 (38.8%) had postoperative complications, including 5 with delayed gastric emptying, 3 with a pancreatic fistula, 2 with a lymphatic fistula, 2 with a biliary tract fistula, and 1 with postoperative atelectasis. Eight participants had infectious complications, including abdominal infection (six cases), wound infection (one case), and pulmonary infection (one case). One of the participants had both a pancreatic fistula and an abdominal infection, and the incidence of severe postoperative complications (Clavien–Dindo classification grade ≥ 3) was 36.8% among all participants with complications. Table 5 shows the basic clinical information of the two groups, and the differences in the days of hospitalization (*p* < 0.001) and hospitalization expenses (*p* < 0.001) were also statistically significant. The PhA value of the participants with complications was significantly lower than those without complications (*p* = 0.013). We constructed a ROC analysis to determine the best PhA cutoff point for predicting complications, and the AUC was 0.717 (*p* = 0.011), as shown in Figure 4a. Participants with a PhA < 5.35 with 66.7% sensitivity and 73.7% specificity (Youden index: 0.404) more easily presented with postoperative complications. The nutritional status assessed by the three common nutrition evaluation tools was not associated with the occurrence of complications by chi-square analysis (Table 6).

In addition, we also analyzed the postoperative complications of the included 74 participants, which included the exclusion of 25 participants due to the diagnosis of other diseases, as presented in the flowchart. A total of 28 (37.8%) participants had postoperative complications and had a significantly lower PhA value (4.99 ± 0.87) than the participants without complications (5.42 ± 0.65, *p* = 0.021). The basic clinical information of the complication and noncomplication groups is presented in Supplementary Table S1. According to the ROC analysis shown in Figure 4b (AUC was 0.659, *p* = 0.022), the best PhA cutoff point for predicting complications of PD among the 74 participants was 4.75 (87.0% sensitivity, 53.6% specificity, Youden index: 0.334).

## 4. Discussion

Pancreatic head cancer has a high rate of cancer-related malnutrition, and almost 80% of patients in terminal states manifest cachexia [18]. It is important to identify pancreatic head cancer patients with malnutrition early and provide nutritional intervention. Our study found that PhA is an objective parameter of BIA that has a good relationship with age, BMI, and nutritional status, and it could be a potential indicator of cancer patients’ nutritional status with a cutoff value of 5.45. In addition, PhA is the only nutrition-related indicator associated with postoperative complications and has a good predictive ability for the incidence of complications in pancreatic head cancer patients who have undergone PD.

Nutritional risk screening and nutritional assessment should be performed immediately after diagnosis for cancer patients. The NRS-2002 and SGA are two recommended nutrition assessment tools that have been widely used in clinical practice. Santos, I.; et al. [19] evaluated 41 pancreatic cancer patients’ nutrition status by the NRS-2002 and patient-generated SGA (PG-SGA). According to the recent GLIM criteria, more than 80% of patients had malnutrition, and 73.2% were diagnosed with malnutrition. The percentage of patients with malnutrition status was higher than our results, which may be caused by the large proportion of stage IV in the former study. However, the current nutrition assessment tools all have the characteristic of subjectivity. The BIA method [20], a non-invasive, rapid, accurate, and practical method for assessing body composition, has been used to evaluate nutrition status.

BIA is an objective method to assess whole-body cell membrane quality and depict fluid distribution for an individual [21]. PhA generated by BIA as the index of cell membrane integrity and vitality has been found to predict malnutrition risk in 122 hospitalized geriatric patients [22], and the cutoff point of PhA for malnutrition risk was 4.7. Although the values of PhA varied among different disease populations, the ability to identify malnutrition has been verified by many studies [23,24]. The lower PhA values of cancer patients presented low muscle mass and muscle abnormalities [25] and represented a clinically feasible approach for the initial identification of malnutrition patients who require nutritional intervention [26]. Regarding the relationship between PhA and subjective nutrition assessment tools, Gupta, D.; et al. [27] discovered that PhA has a good relation with SGA among advanced colorectal cancer patients. In addition, NRS-2002 [28] and GLIM [29] were also significantly correlated with PhA in different diseases. Our study was the first to find that PhA was associated with NRS-2002, SGA, and GLIM in pancreatic head cancer patients through Spearman correlation analysis, which also suggested that PhA is a potential nutritional indicator in pancreatic cancer. In addition, our study also determined that the cutoff point of PhA for malnutrition diagnosis was 5.45.

However, the normal range of PhA and the cutoff point for predicting malnutrition are still controversial because the value is affected by age, sex, and BMI [30]. Our results agreed with this finding because the value of PhA was negatively correlated with age and positively correlated with BMI. In addition, all studies used different cutoff values for PhA [31,32], perhaps to account for the pathophysiology of the disease, which may have different effects on cell membrane integrity and cellular hydration. Thus, the PhA values that are used to evaluate nutrition status may also differ between groups of patients with different clinical conditions.

Nutrition status has a major impact on surgery complications, especially in cancer patients. A prospective study [33] collected 355 patients who underwent PD and evaluated nutritional status by mini nutrition assessment (MNA), and compared with well-nourished patients, patients with nutritional issues showed a higher risk of complications. Additionally, the nutrition status assessed by the PG-SGA [34] and NRS-2002 [35] was also closely related to the postoperative complications of patients with ampullary carcinoma after PD. In contrast, Probst et al. studied 12 nutritional assessment tools and found that none of these tools defined malnutrition as relevant to complications after pancreatic surgery [36]. Our results also show that the malnutrition risk assessed by the NRS-2002, SGA, and malnutrition diagnosed by the GLIM criteria were unrelated to postoperative complications, which may be caused by preoperative nutritional support for patients at nutritional risk. Therefore, these nutritional assessment tools can be used to identify patients’ nutrition status, but their predictive value with regard to complications needs further study.

Not much research exists on the PhA value for predicting complications in cancer patients. Lundberg, M.; et al. [37] found that the median PhA of 61 head neck cancer patients was 4.5, and low PhA was associated with a higher surgical complication rate and a longer hospital stay. In advanced epithelial ovarian cancer, low PhA was an independent predictor of perioperative complications [38]. Our results agreed that a low PhA was closely associated with complications and the cutoff point was 5.35°. A recent study [39] proposed that PhA on postoperative day (POD) 2 below the 5th percentile had higher local complication rates. We also performed BIA on POD 7 and recorded the PhA value. Although the PhA value of the complication group (4.42 ± 0.67) was significantly lower than that of the non-complication group (4.99 ± 0.69, *p* = 0.033), Spearman correlation analysis found that PhA was not associated with complications (*p* = 0.052). This is probably caused by the perioperative fluid administration that may influence the intracellular and extracellular fluid distribution, which can alter the PhA value for a short time. Notably, several factors [40] have previously been reported to be independent risk factors for complications after PD, such as being elderly, male, and having a BMI higher than 40 kg/m^2^, severe comorbidities, and sarcopenia [41]. However, these factors were not associated with postoperative complications in the present study. This may be due to the different patient characteristics and treatment regimens.

In addition, some scholars recommend using a standardized phase angle (SPA) that is calculated by observed PhA and a reference PhA value that has been adjusted according to age, sex, and BMI [42]. Among the published reference PhA values for healthy populations [30,43], only the reference values generated from the German population [30] are stratified according to sex, age, and BMI. The current research [44,45] related to SPA is based on the reference value generated from the German population. However, we cannot determine the corresponding SPA value due to the lack of reference PhA values of large sample data generated from the Chinese population. Therefore, it is important to set up and compare the reference PA value from a different population.

There are some limitations in this study. The sample size was not very large due to the strict entry criteria, so we could not analyze each cancer stage separately, and this can only be achieved by increasing the sample size. In addition, survival time is an important prognostic parameter for cancer patients. Many studies support that a low PhA predicts poor prognosis in cancer patients and put up many different cutoff values, so our study did not duplicate these results. Although the results showed that low PhA was significantly related to malnutrition and a high complication rate, it may apply to the Asian population but not to individuals from other countries because PhA values differ according to the population.

## 5. Conclusions

Our results show that PhA as the subjective parameter of BIA was associated with nutrition status and postoperative complications among pancreatic head cancer patients who underwent PD. Further studies should focus on the effectiveness of PhA for assessing the perioperative nutritional interventions to improve outcomes.

## Figures and Tables

**Table 1:** Patient characteristics at baseline.

Variable	Female	Male	*p*-Value
	*n* = 18	*n* = 31	
Age (years)	57.61 ± 10.21	58.35 ± 11.98	0.826
BMI (kg/m^2^)	21.81 ± 2.77	22.35 ± 3.17	0.549
PhA (°)	4.97 ± 0.70	5.44 ± 0.73	0.035
Total Body Water (kg)	27.73 ± 4.68	35.75 ± 4.52	<0.001
Skeletal Muscle (kg)	20.41 ± 3.78	27.02 ± 3.83	<0.001
Fat Free Mass (kg)	37.78 ± 6.35	48.62 ± 6.14	<0.001
Fat Mass (kg)	17.61 ± 6.04	16.25 ± 6.75	0.485
Biceps circumference (cm)	27.77 ± 2.72	29.46 ± 3.31	0.072
ALB (g/L)	40.78 ± 3.95	41.29 ± 5.00	0.711
Days of Hospitalization (days)	25.78 ± 15.99	25.71 ± 11.80	0.068
Hospitalization Costs (CNY)	99,665.67 ± 39,848.01	93,999.74 ± 21,012.52	0.157

**Table 2:** Spearman correlation between PhA and clinical parameters.

Variable	Spearman Correlation Coefficient	*p*-Value
Age (years)	−0.398	0.005
BMI (kg/m^2^)	0.325	0.023
Biceps circumference (cm)	0.422	0.003
ALB (g/L)	0.331	0.020
Days of Hospitalization (days)	−0.146	0.317
Hospitalization Costs (CNY)	−0.226	0.119

**Table 3:** Comparison of PhA values between the malnourished and well-nourished groups.

Items	Malnourished	Well Nourished	*p*-Values
SGA	5.07 ± 0.71	5.53 ± 0.73	0.032
NRS-2002	5.09 ± 0.70	5.53 ± 0.75	0.043
GLIM	4.94 ± 0.61	5.53 ± 0.76	0.005

**Table 4:** Spearman correlation between PhA and nutritional status.

Variable	Spearman Correlation Coefficient	*p*-Value
SGA	0.314	0.028
NRS-2002	0.312	0.029
GLIM	0.421	0.003

**Table 5:** The basic clinical data for participants with and without complications.

Variable	Complications	No Complications	*p*-Values
	*n* = 19	*n* = 30	
Age (years)	57.63 ± 11.79	58.37 ± 11.10	0.826
PhA (°)	4.94 ± 0.67	5.47 ± 0.73	0.013
BMI (kg/m^2^)	22.06 ± 2.99	22.21 ± 3.08	0.868
Total body water (kg)	32.85 ± 4.81	32.77 ± 6.69	0.965
Skeletal muscle (kg)	24.47 ± 3.97	24.66 ± 5.56	0.900
Fat-Free Mass (kg)	44.66 ± 6.51	44.62 ± 9.08	0.985
Fat Mass (kg)	17.02 ± 8.22	16.58 ± 5.21	0.820
Biceps circumference (cm)	28.79 ± 3.12	28.87 ± 3.28	0.930
ALB (g/L)	39.89 ± 5.05	41.87 ± 4.21	0.146
Days of Hospitalization (days)	35.84 ± 16.00	19.33 ± 5.06	<0.001
Hospitalization Expenses (CNY)	119,924.05 ± 32,990.61	80,974.57 ± 12,017.24	<0.001

**Table 6:** Chi-square test results for the correlation between nutritional status and complications.

Items	Malnutrition	Complications in Malnutrition Group	χ^2^-Value	*p*-Value
NRS-2002	29	12	0.203	0.652
SGA	28	13	1.612	0.204
GLIM	22	10	0.750	0.386

## Data Availability

The data presented in this study are available on request from the corresponding author.

## Supplementary Materials

The following supporting information can be downloaded at: https://www.mdpi.com/article/10.3390/ijerph19116426/s1, Table S1: The basic clinical data of 74 participants with and without complications.
