# Supplementary material for: The Relationship between Phase Angle, Nutrition Status, and Complications in Patients with Pancreatic Head Cancer
Source: Int J Environ Res Public Health. 2022 May 25;19(11):6426. doi: 10.3390/ijerph19116426 (PMC9180801; doi:10.3390/ijerph19116426)
Supplement: Supplementary file 1 [file ijerph-19-06426-s001.zip › ijerph-1682372-supplementary.pdf]

Table S1. The basic clinical data of 74 participants with and without complications.

Variable	Complications N = 28	No Complications N = 46	*P*-Values
Age (years)	59.75 ± 11.22	57.78 ± 10.20	0.441
PhA (°)	4.99 ± 0.87	5.42 ± 0.65	0.021
BMI (kg/m^2)	22.65 ± 3.17	22.87 ± 3.42	0.780
Skeletal muscle (kg)	29.28 ± 3.17	29.38 ± 3.28	0.904
Fat Free Mass (kg)	45.94 ± 7.52	44.71 ± 8.78	0.538
Fat Mass (kg)	17.90 ± 8.27	17.97 ± 6.57	0.969
Biceps circumference (cm)	25.23 ± 4.63	24.64 ± 5.32	0.624
ALB (g/L)	40.43 ± 5.03	42.30 ± 3.40	0.080
Days of Hospitalization (days)	33.93 ± 13.79	18.59 ± 4.30	< 0.001
Hospitalization Expenses (CNY)	118906.26 ± 41475.25	73782.98 ± 15602.92	< 0.001
